# ID 295 - Projeto Nats Itinerante, o Nats-HUJM mais perto de você!

**DOI:** 10.5327/2237-9622.2025.v34s1.295

**Published:** 2025-11-25

**Authors:** Jéssica Weis Bonfanti, Helder Cassio de Oliveira, Letícia Rossetto da Silva Cavalcante

## Abstract

**Introdução:**

Os Núcleos de Avaliação de Tecnologias em Saúde (Nats) foram instituídos nos hospitais de ensino de todo o País com o intuito de introduzir a cultura de ATS nos hospitais. O Nats é o responsável por realizar ATS por meio da utilização de evidências científicas capazes de auxiliar o gestor hospitalar na tomada de decisão quanto à incorporação, alteração ou exclusão de tecnologias, bem como promover o uso apropriado e a racionalidade técnica na alocação de recursos. Além disso, possui o papel fundamental de introduzir e promover a cultura da Prática em Saúde Baseada em Evidências (PSBE) na rotina dos profissionais de saúde. No entanto, a ATS ainda não se apresenta como uma atividade que faça parte da gestão hospitalar atualmente. A falta de cultura de ATS nas instituições gera um grande obstáculo, pois os profissionais da saúde, de maneira geral, desconhecem o tema, mesmo após todos os anos de esforços empreendidos para sua disseminação. Sendo assim, a criação de um Nats não constitui ação suficiente para a difusão da cultura de ATS, havendo fragilidade na influência do núcleo no processo de tomada de decisão clínica e gerencial em hospitais. Devido a todos esses desafios da ATS hospitalar, foi desenvolvido o projeto do Nats Itinerante. Essa ação faz parte de um projeto chamado "Desenvolvimento da cultura de avaliação de tecnologias em saúde entre profissionais e gestores de um hospital universitário", que visa ao desenvolvimento de ações que estimulem a cultura do uso da ATS pelos profissionais do Hospital Universitário Júlio Müller (HUJM) na tomada de decisão. O objetivo do Nats Itinerante foi oferecer um momento de troca de conhecimentos com os profissionais em seus locais de trabalho, divulgando informações básicas e de fácil compreensão sobre tecnologias em saúde, ATS, as funções do Nats e a forma de solicitar os serviços do Nats do nosso hospital. Todas essas informações foram compiladas por meio da elaboração de um vídeo de 3min37s, realizado com o auxílio de um profissional da Unidade de e-Saúde. Foi estabelecido um cronograma de locais a serem visitados, sendo sua divulgação realizada previamente nos grupos de mensagens instantâneas institucionais. Em torno de duas a quatro pessoas envolvidas com o Nats-HUJM participavam dessas visitas, iniciando com a apresentação das informações por meio de um vídeo e, após, realizando uma troca de ideias com os participantes. Ao final da discussão, todos os participantes ganhavam uma "lembrancinha" (bombom) com o seguinte agradecimento: "Obrigada por fazer parte do Nats Itinerante! É o Nats-HUJM mais perto de você!". Assim, 413 pessoas de 37 setores/unidades em 3 turnos diferentes participaram dessa ação. O vídeo foi apresentado em torno de 74 vezes em 9 dias de projeto. Para o presente congresso, a apresentação desta proposta será por meio de comunicação oral expositiva com projeção de multimídia.

**Método:**

A apresentação acontecerá em três etapas. Na primeira etapa, será apresentada brevemente a justificativa do projeto. Após, será exibido um vídeo de 3min37s, que foi utilizado para a disseminação das informações sobre ATS para os profissionais do hospital. Por último, serão demonstrados os resultados do projeto, contando com a utilização de imagens, sendo necessário monitor, equipamento de som e passador de eslaides com .

